# Eye Gaze in Autism Spectrum Disorder: A Review of Neural Evidence for the Eye Avoidance Hypothesis

**DOI:** 10.1007/s10803-022-05443-z

**Published:** 2022-02-04

**Authors:** Nicole Stuart, Andrew Whitehouse, Romina Palermo, Ellen Bothe, Nicholas Badcock

**Affiliations:** 1 University of Western Australia, 35 Stirling Highway, Crawley, WA 6009 Australia; 2 Telethon Kids Institute, Perth Children’s Hospital, 15 Hospital Avenue, Nedlands, WA 6009 Australia

**Keywords:** Autism spectrum disorder, Eye gaze, Eye avoidance, Amygdala, Social brain

## Abstract

Reduced eye contact early in life may play a role in the developmental pathways that culminate in a diagnosis of autism spectrum disorder. However, there are contradictory theories regarding the neural mechanisms involved. According to the amygdala theory of autism, reduced eye contact results from a hypoactive amygdala that fails to flag eyes as salient. However, the eye avoidance hypothesis proposes the opposite—that amygdala hyperactivity causes eye avoidance. This review evaluated studies that measured the relationship between eye gaze and activity in the ‘social brain’ when viewing facial stimuli. Of the reviewed studies, eight of eleven supported the eye avoidance hypothesis. These results suggest eye avoidance may be used to reduce amygdala-related hyperarousal among people on the autism spectrum.

Autism spectrum disorder (ASD) is a complex neurodevelopmental disorder, characterised by restricted interests and deficits in social interaction and social communication. A wide array of genetic and neurobiological factors has been associated with ASD, varying enormously between individuals (Constantino et al., 2021; Johnson et al., 2021; Klin et al., 2020). However, recent developmental studies suggest these heterogeneous vulnerabilities may converge on common endophenotypes, which appear prior to diagnosis and play a role in the development of ASD (Constantino et al., 2021). Of these, one of the most replicable is reduced eye contact, with many arguing that reduced gaze to the eyes of others plays a role in the development of ASD (Klin et al., 2020). However, there is conflicting evidence and contradictory theories as to the mechanisms and brain regions involved. Where the amygdala theory of autism proposes that reduced eye contact results from a hypoactive amygdala, which reduces the innate salience of eyes in social and communication development (Baron-Cohen et al., 2000), the eye avoidance hypothesis suggests the opposite—that amygdala hyperactivity causes unpleasant levels of arousal and this unpleasant arousal leads to eye avoidance (Tanaka & Sung, 2013). The current review examines literature that measured activity in brain areas associated with processing social stimuli, with a particular focus on the amygdala, in an attempt to determine the neural mechanisms underpinning reduced eye contact in ASD.

## A Developmental Account of ASD

Longitudinal studies indicate that ASD’s behavioural and social-communicative features start to emerge from the second year of life, although neurobiological differences are apparent from as early three months (Bosl et al., 2018; Landa et al., 2013; Ozonoff et al., 2010; Varcin & Nelson, 2016; Wang et al., 2018). This evidence suggests the behaviours characteristic of ASD may be the culmination of an underlying developmental process or processes, in which molecular and neurobiological liabilities interact with the environment in an iterative manner over the first three years of life (Constantino et al., 2021; Johnson et al., 2005, 2021; Jones et al., 2014; Klin et al., 2020; Shultz et al., 2018; Tiede & Walton, 2020). By the age of one, the typically developing brain has doubled in size and synaptic density has quadrupled (Petanjek et al., 2011; Pfefferbaum et al., 1994). Rapid brain growth during this period is not only guided by genetics, but also by epigenetic processes shaped by a child’s interactions with the environment (Shultz et al., 2018; Szyf & Bick, 2013). In typical development, progressively more complex social interactions are scaffolded on previous social learning, driving development of the social brain (Constantino et al., 2021; Klin et al., 2020). However, small genetic susceptibilities early in life can alter an infant’s interaction with the environment, augmenting neural and behavioural differences, with a cascading influence on social development (Klin et al., 2020; Shultz et al., 2018; Whitehouse et al., 2021).

While there is considerable variability in phenotypic expression at the point of ASD diagnosis (Klin et al., 2020), there is emerging evidence for homogeneity in early development mechanisms that lead to ASD expression. (Constantino et al., 2021). Recent empirical data and theoretical accounts suggest that widely varying neurobiological and genetic liabilities converge upon a discrete number of endophenotypes, which play a role in ASD’s development (Constantino et al., 2021). If this is the case, then understanding these endophenotypes is critical to understanding how ASD develops and also how the difficulties which impact quality of life for people on the autism spectrum may be effectively treated or prevented.

## The Role of Eye Gaze in the Development of ASD

A number of endophenotypes are potentially linked to the development of ASD, including differences in attentional disengagement, motor delays and sensory disturbances (Constantino et al., 2021; Johnson et al., 2015, 2021; Klin et al., 2020; Tiede & Walton, 2020; Varcin & Nelson, 2016). However, the most reliably replicated early predictor of ASD is differences in social attention, and in particular, attenuated eye gaze (Klin et al., 2020; Tiede & Walton, 2020).

Reduced eye gaze as a potential endophenotype to clinical ASD has received great interest over many decades (Itier & Batty, 2009; Klin et al., 2020; Phillips et al., 1992; Schultz, 2005; Tiede & Walton, 2020). Human babies have an innate preference for faces (Goren et al., 1975; Valenza et al., 1996) and the eye region in particular draws their attention (Batki et al., 2000). Relative to typically developing children, however, infants later diagnosed with ASD look less at faces and show reduced eye contact and gaze-following behaviour (Leekam et al., 1998; Merin et al., 2007; Riby et al., 2009). In fact, reduction in eye gaze over the first six months of life is correlated with the severity of later social difficulties (Jones & Klin, 2013). Eye gaze is likely to be critical for the development of social skills and higher order social-cognitive abilities, such as theory of mind and perspective taking (refer Stephenson et al., 2021 for a review). Eyes are an important source of social information. Indeed, eye contact improves the ability to infer mental states (Adams & Nelson, 2016). It is also critical for initiation of joint attention (Hamilton, 2016)—allowing people to share experiences. Importantly, eye contact signals a desire to interact with others (Mundy & Newell, 2007)—increasing opportunities for interaction, affiliation and social learning.

Consistent evidence supports the role of eye gaze in social development in both clinical and neurotypical populations throughout the lifespan. Gaze-following and use of eye contact to establish social interaction in infancy positively correlates with later acquisition of social cognition and social skills (Vaughan Van Hecke et al., 2007). Interestingly, a recent review has provided evidence that children with visual impairment experience language, communication and social difficulties during the second and third year of life and a disproportionate number of these children also exhibit stereotyped behaviours (Vervloed et al., 2020). It is possible that social difficulties in children with visual impairment are caused by reduced access to visual sources of social information. Eye contact also supports processing of social information and social skills during adulthood. For example, duration of eye contact in real life situations positively correlates with adult social skills and emotion recognition accuracy (Cherulnik et al., 1978; Hall et al., 2010), and eye gaze is also linked to better face identity memory (Davis et al., 2017). Meanwhile, studies of adults on the autism spectrum have found an association between reduced visual fixation on the eye region and greater social difficulties (Jones et al., 2008; Speer et al., 2007).

While the role of eye gaze in the development of ASD has received much attention, there are conflicting theories on the mechanisms responsible for differences in eye contact. A prominent explanation is that social stimuli, and especially the faces and eyes of others, are less salient for people on the autism spectrum (Baron-Cohen et al., 2000; Klin et al., 2002; Weeks & Hobson, 1987) because they are less rewarding (Chevallier et al., 2012; Grelotti et al., 2002) and/or less informative (Baron-Cohen et al., 1997; Grelotti et al., 2002). The amygdala theory of autism is a brain-based theory that claims people on the autism spectrum perceive faces and eyes as less salient relative to neurotypical people due to amygdala hypoactivation (Baron-Cohen et al., 2000). However, a more recent hypothesis challenges this theory. In 2013, Tanaka and Sung reviewed evidence of face processing difficulties in ASD and proposed the “eye avoidance hypothesis” of autism. According to this hypothesis, people on the autism spectrum experience high levels of unpleasant amygdala-mediated arousal in response to direct eye contact and use eye avoidance as a strategy to reduce this arousal (Tanaka & Sung, 2013). The remainder of this review provides an overview of these competing theories and seeks to arbitrate between them by reviewing studies that specifically measured the relationship between eye gaze and brain activity in ASD.

## The Amygdala Theory of Autism

The amygdala is a subcortical brain region involved in rapid and non-conscious detection of stimuli with biological, social, and emotional salience (Zalla & Sperduti, 2013). Magnocellular retinal ganglion cells convey biologically important information to the amygdala via the superior colliculus and pulvinar nucleus of the thalamus (Méndez-Bértolo et al., 2016). This subcortical pathway is involved in detection of faces, especially direct eye contact (Johnson, 2005; Senju & Johnson, 2009; Skuse, 2003), and is implicated in infant face preferences (Johnson, 2005). The amygdala and related subcortical regions may be especially important for threat detection, including rapid detection of emotional expressions, especially fear (McFadyen et al., 2019; Méndez-Bértolo et al., 2016). The amygdala, in particular, drives arousal responses to emotionally salient and threatening stimuli (Rodriguez-Romaguera et al., 2020). Importantly, activity in the amygdala modulates cortical regions involved with the interpretation of social information (Hadjikhani et al., 2017a, 2017b; Senju & Johnson, 2009). Given its central role in processing social and emotional stimuli, the amygdala has been the focus of a great deal of research on the neurobiological underpinnings of ASD.

We do not provide an exhaustive review of evidence supporting the amygdala theory of autism in the current paper, as this has been done elsewhere (refer Sweeten et al., 2002). Briefly, advocates of the amygdala theory argue that people on the autism spectrum exhibit a similar profile of difficulties to people with amygdala lesions. Neurotypical participants have a tendency to focus on the eye region when asked to make judgements about faces; for example, judgements of emotion or gender (Peterson & Eckstein, 2011). However, people with amygdala damage (Schyns et al., 2005) and people on the autism spectrum (Papagiannopoulou et al., 2014; Pelphrey et al., 2002; Spezio et al., 2007) spend less time looking at the eyes relative to controls. Moreover, people on the autism spectrum and people with amygdala damage have more difficulty in face tasks that rely on information from the eye region, such as recognising complex mental states (amygdala damage: Adolphs et al., 2002; Tranel et al., 1994; ASD: Baron-Cohen et al., 1997) and negative emotions (amygdala damage: Anderson et al., 2000; ASD: Ashwin et al., 2006), especially fear (amygdala damage: Adolphs et al., 2002; Broks et al., 1998; Calder, 1996; Schyns et al., 2005; Tranel et al., 1994; ASD: Howard et al., 2000; Uljarevic & Hamilton, 2013).

Differences in amygdala activation are also cited as evidence supporting the amygdala theory of autism. In neurotypical individuals, activity in the amygdala increases in response to eye contact (Skuse, 2003) and emotional expressions (Wright et al., 2002), particularly those that are negative (Morris et al., 1996). However, there is evidence of amygdala hypoactivation during face processing tasks in people on the autism spectrum (Baron-Cohen et al., 1999; Critchley et al., 2000). Indeed, a recent meta-analysis of whole brain fMRI studies concluded that the primary difference between participants on the autism spectrum and neurotypical controls during face processing tasks was reduced amygdala activation in the participants diagnosed with ASD (Costa et al., 2021).

## The Current Review

While there is substantial evidence in favour of the amygdala theory of autism, support is building for the reverse hypothesis—that amygdala hyperactivity is responsible for eye avoidance in ASD. Recently, a systematic review investigated whether hypoarousal or eye avoidance was responsible for emotion recognition difficulties in ASD (Cuve et al., 2018). This review found mixed evidence that reduced eye gaze is associated with emotion recognition impairments in ASD. Furthermore, there was no consistent evidence supporting either hypoarousal or hyperarousal during emotion processing tasks. The review identified studies that investigated emotion recognition impairments using both eye-tracking and neurophysiological measures (Corden et al., 2008; Dalton et al., 2005; Hubert et al., 2009; Kliemann et al., 2012; Mathersul et al., 2013; Zürcher, Donnelly, et al., 2013; Zürcher, Rogier, et al., 2013). Of these, three found evidence of hypoarousal and three found evidence of hyperarousal. Although, Cuve et al. (2018) mention the possibility that eye gaze may modulate arousal, their review did not attempt to disentangle this relationship. Specifically, it is possible that previous findings were contradictory as they did not control for gaze. As such, the current review assesses studies that measured the relationship between eye gaze and neural activity in ASD.

In particular, the current review asked the following research questions. Firstly, does eye gaze affect the localisation and magnitude of brain responses to facial stimuli in ASD and, secondly, what are the differences in localisation and magnitude of brain activity between neurotypical people and people on the autism spectrum when eye gaze is controlled. All studies met the following inclusion criteria:

1. Peer reviewed study, published in English;
2. Use of groups of neurotypical controls and participants diagnosed with ASD;
3. Use of passive face viewing or active face processing tasks;
4. Manipulated or directed attention to the eye region and/or measured fixation on the eye region using eye tracking; and
5. Measured neural responses to facial stimuli using fMRI.

It is important to note that neuroimaging studies focusing on regions of interest (ROIs), rather than whole brain activation patterns, have been criticised for artificially inflating group differences and for poor replicability (Gentili et al., 2019, 2021). To date, however, no study has compared whole brain responses to facial stimuli in free-viewing and gaze-cuing conditions. Such studies are necessary to understand the causal role that eye gaze may play in neural activation in ASD. Therefore, ROI analyses were excluded from the review unless they specifically compared neural responses in free-viewing and gaze-cuing conditions. Eight whole brain and three ROI studies were identified that met the study’s inclusion criteria (refer Table 1).

**Table 1:** Studies included in the review

Author (Date)	Sample	ASD Severity	IQ ASD Group	Gender Breakdown	Age	Independent Variables	Dependent Variables	Visual Stimuli	fMRI task	Gaze Manipulation or Measure
Dalton et al. (2005) Study 1	14 participants diagnosed with autism or Asperger’s disorder; 12 age matched controls	All participants were verbally fluent	Full Scale IQ: M = 94, SD = 19.47	All males	ASD: M = 15.9, SD = 4.71; Controls: M = 17.1, SD = 2.78	ASD diagnosis; emotional expression of facial stimuli; gaze direction of facial stimuli	Whole brain activation patterns; emotion recognition accuracy & response time; time spent fixating the eyes	Black & white photographs of emotional (fearful, happy, angry) & neutral faces with averted & direct gaze from the Karolinska Directed Emotional Faces. Stimuli presented for 3 s	Facial emotion discrimination	Eye tracking used to measure eye movements & fixations
Dalton et al. (2005) Study 2	16 participants diagnosed with autism or Asperger’s disorder; 16 age matched controls	2 participants had minimal functional speech; 14 participants were verbally fluent	Full Scale IQ: M = 92.1, SD = 27.7	All males	ASD: M = 14.5, SD = 4.60; Controls: M = 14.5, SD = 4.56	ASD diagnosis; familiarity of facial stimuli	Whole brain activation patterns; time spent fixating the eyes	10 photographs of the participants’ family members or friends; 10 photographs of other participants’ family members & friends	Face recognition	Eye tracking used to measure eye movements & fixations
Dapretto et al. (2006)	10 participants diagnosed with ASD; 10 age, gender and IQ matched controls	ADOS-G Social Subscale: M = 8.5, SD = 3.0; ADI-R Social Subscale: M = 20.3, SD 4.9	Full Scale IQ: M = 96.4, SD = 18.3	ASD (1 female, 9 males); Controls (1 female, 9 males)	ASD: M = 12.0, SD = 2.5; Controls: M = 12.4, SD = 2.2	ASD diagnosis; imitation of facial emotion; observation of facial emotion	Whole brain activation patterns	80 faces expressing 5 different emotions: anger, fear, happiness, neutrality or sadness. Stimuli presented for 2 s	Imitation and passive observation of facial expressions	Fixation cross used to cue attention to the eyes. Eye tracking used as a manipulation check
Davies et al. (2011)	16 children diagnosed with ASD; 16 age, gender & IQ matched controls	ADOS-G: M = 12, SD = 4.0; ADI-R: M = 21.53, SD = 7.7	Verbal IQ: M = 100.38, SD = 19.9; Performance IQ: M = 111.13, SD = 19.83; Full Scale IQ: M = 106.19, SD = 20.31	ASD (2 females, 14 males); Controls: (2 females, 14 males)	ASD: M = 11.69, SD = 2.71; Controls: M = 12.30, SD = 1.88	ASD diagnosis; gaze direction of face stimuli	Whole brain activation patterns	Photographs of 160 faces with different expressions (anger, fear, happiness, neutral). 50% averted gaze & 50% direct gaze. Stimuli presented for 2 s	Passive viewing	Fixation cross used to cue attention to the eyes; eye tracking used as a manipulation check
Hadjikhani et al. (2017a, 2017b)	23 participants diagnosed with ASD; 20 age & IQ matched controls	AQ: M = 26.8, SEM = 1.4	Full Scale IQ: M = 112.9, SEM = 3.3	ASD (2 females, 21 males); Controls (3 females, 17 males)	ASD: M = 22.6, SEM = 1.8; Controls: M = 23.3, SEM = 1.8	ASD diagnosis; emotional expression of facial stimuli; visual cueing to the eye region	ROI activation (left & right amygdala; superior colliculus; pulvinar nucleus of the thalamus)	24 movies created from the NimStim database. Movies showed morphs of facial expressions from neutral to fear, happiness or anger. Movies were 5 s each	Passive viewing	Fixation cross used to cue attention to the eyes
Kliemann et al. (2012)	16 participants diagnosed with ASD; 17 controls	AQ: M = 36, SD = 1.85	Vocabulary IQ: M = 108, SD = 7.38; Strategic thinking IQ: M = 128, SD = 10.82	All males	ASD: M = 30.44, SD = 6.34; Controls: M = 30.47, SD = 6.24	ASD diagnosis; initial fixation; emotional expression of facial stimuli	Emotion recognition accuracy & response time; eye movements away from or towards the eye region of facial stimuli; ROI activation (amygdala)	120 greyscale faces displaying happy, fearful & neutral expressions presented for 150 ms	Emotional classification task	Fixation cross used to cue attention to the eyes or mouth; eye tracking to measure eye movements
Lassalle et al. (2017)	27 participants diagnosed with ASD; 21 controls	ADOS-G: M = 6.32, SD = 1.57; ADI-R: M = 41.61, SD = 7.80	Full Scale IQ: M = 113.15, SD = 12.36	All males	ASD: M = 23.63, SD = 9.86; Controls: M = 19.70, SD = 7.74	ASD diagnosis; emotional expression of facial stimuli	Whole brain activation patterns; ROI activation (amygdala, vmPFC, ACC, right STS/TPJ, & FFA)	8 faces from the MacBrain Face Stimulus set; 4 fearful, angry, happy, & neutral expressions for each face. Stimuli presented for 300 ms	Passive viewing	Fixation cross used to cue attention to the eyes
Perlman et al. (2011)	12 participants diagnosed with autism; 7 age & verbal IQ matched controls	ADI-R Social: M = 21.6, SD = 3.2; ADI-R Communication Verbal: M = 16.5, SD = 4.1; ADI-R Communication Nonverbal: M = 9.0, SD = 3.0; ADI-R Stereotyped Behaviours: M = 6.1, SD = 2.4; ADOS-G Communication: M = 4.8, SD = 1.2; ADOS-G Social: M = 8.5, SD = 2.8; ADOS-G Stereotyped Behaviours: M = 1.4, SD = 1.3	Verbal IQ: M = 106.7, SD = 11.7; Performance IQ: M = 102.2, SD = 15.8	ASD (1 female, 11 males); Control (7 males)	ASD: M = 25.5, SD = 7.47; Controls: M = 28.57, SD = 5.74	ASD diagnosis; visual scanpath	Whole brain activation patterns and ROI activation (amygdala & FFA). ROIs defined based on whole brain differences between NT and ASD participants during free viewing of facial stimuli	A full colour fearful, male face from the NimStim set of facial expressions, presented for 12 s	Passive viewing	Visual scanpaths defined by a moving crosshair
Tottenham et al. (2014)	28 participants diagnosed with ASD; 42 controls	AQ: M = 34, SD not reported	Full Scale IQ: M = 103, SD not reported	ASD (3 females, 25 males); Controls (12 females, 30 males)	ASD: M = 16, SD = 7; Controls: M = 17, SD = 8	ASD diagnosis; visual cueing to the eye region; emotional expression of facial stimuli	Facial threat ratings; emotion recognition accuracy; eye movements towards the eye region of facial stimuli; ROI activation (amygdala)	18 greyscale images of facial expressions (angry, neutral, happy) presented for 300 ms	Passive viewing	Visually degraded geometric shape used to cue attention to the eye region; eye tracking used as a manipulation check
Zürcher et al. (2013a, 2013b)	16 participants diagnosed with ASD; 18 age & IQ matched controls	ADI-R Social: M = 20.67, SD = 3.94; ADI-R; Communication: M = 12.93, SD = 4.20; ADI-R Stereotypies: M = 4.27, SD = 1.83; ADI-R Development: M = 2.93, SD = 1.44; ADOS-G Communication: M = 4.00, SD = 1.37; ADOS-G Social: M = 7.88, SD = 2.47; AQ**:** M** = **30.46, DS = 4.6)	Performance IQ: M = 108.7, SD = 13.3	ASD (3 females, 13 males); Controls: (2 females, 16 males)	ASD: M = 23.5, SD = 6.8; Controls: M = 25.8, SD = 5.3	ASD diagnosis; visual cueing to the eye or mouth region; facial stimuli inverted or upright	Thatcherised stimuli discrimination accuracy; whole brain activation patterns; ROI activation (FFA; lateral occipital cortex; IFG; amygdala; pulvinar nucleus of the thalamus)	Upright or inverted pairs of facial stimuli (face with Thatcherised eyes vs. typical face; face with Thatcherised mouth vs. typical face; face with both features Thatcherised vs. typical face). Stimuli pairs presented for 1350 ms	Thatcherised face discrimination	Verbal cues to attend to changes at the eyes or the mouth
Zürcher et al. (2013a, 2013b)	22 participants diagnosed with ASD; 22 controls	AQ: M = 28, SD = 7.0	Performance IQ: M = 114, SD = 15	ASD (3 females, 19 males); Controls (3 females, 19 males)	ASD: M = 27.6, SD = 7.7; Controls: M = 23.7, SD = 5.9	ASD diagnosis; gaze direction of face stimuli	Whole brain activation patterns; ROI activation (thalamus, amygdala & superior colliculus)	8 greyscale fearful faces with averted or direct gaze from the NimStim Set of Facial Expressions database. Stimuli presented for 300 ms	Passive viewing	Fixation cross used to cue attention to the eyes; eye tracking used as a manipulation check

*ASD* Autism Spectrum Disorder, *ADOS-G* Autism Diagnostic Observation Schedule—Generic, *ADI-R* Autism Diagnostic Interview—Revised, *AQ* Autism Spectrum Quotient, *ROI* Region of Interest, *ACC* anterior cingulate cortex, *FFA* fusiform face area, *IFG* inferior frontal gyrus, *STS/TPJ* superior temporal sulcus/temporal parietal junction, *vmPFC* ventromedial prefrontal cortex

The following sections provide an analysis of key findings from these studies in relation to the amygdala, as well neural responses in the “social brain”—a network of brain regions involved in processing social information in humans (Senju & Johnson, 2009). Unless stated otherwise, the studies reviewed use images of faces with direct gaze.

## Eye Gaze and Amygdala Activity

Dalton et al. (2005) were among the first to investigate the relationship between gaze and neural activity in ASD. They assessed neural responses in participants on the autism spectrum and neurotypical controls across two studies using emotion discrimination and face recognition tasks. In both studies, participants on the autism spectrum spent less time fixating the eye region than control participants. Furthermore, fixation time to the eye region was strongly positively correlated with amygdala activity in participants on the autism spectrum, but there was no evidence of a correlation in neurotypical controls. The authors concluded that eye contact was associated with amygdala-mediated emotional arousal in ASD. As a correlational analysis, however, they were unable to provide evidence of causality. Specifically, did eye gaze increase amygdala activity, did amygdala activity increase eye gaze, or did a third variable influence eye gaze and amygdala activity?

Subsequently, a number of studies investigated neural activity in ASD while cuing gaze to the eye region of faces.^1^ Lassalle et al. (2017) used a fixation cross overlaid on facial stimuli to direct gaze to the eye region of neutral and emotional faces. They found that participants on the autism spectrum had greater activity than neurotypical controls in a range of brain areas, including the amygdala, for low-intensity fearful faces, but less activity for happy faces. Meanwhile, Zürcher et al. (2013a, 2013b) compared responses to fearful faces with averted or direct gaze in participants on the autism spectrum and neurotypical controls. Like Lassalle et al., Zürcher et al., used a fixation cross to direct attention to the eye region of facial stimuli. Unlike neurotypical controls, participants on the autism spectrum had greater activity in the subcortical pathway (superior colliculus and thalamus, but not amygdala) for faces with direct, rather than averted gaze—indicating an overactive arousal response to fearful faces with direct gaze (Zürcher et al., 2013a, 2013b). However, participants on the autism spectrum showed attenuated activation of the subcortical pathway for fearful faces with averted gaze compared to control participants. Given fearful faces with averted gaze can signal an environmental threat, this hypoactivity may reflect difficulties with joint attention. Zürcher et al. speculated that people on the autism spectrum lack sensitivity to implicit social cues of threat.

While these two studies provide evidence of subcortical hyperactivity when people on the autism spectrum process fearful faces with direct gaze, they could not determine if eye gaze played a causal role in this hyperactivity because they did not include a free viewing control condition. In order to determine whether eye gaze plays a causal role in generating amygdala activity in ASD, a number of studies have compared amygdala activity during free viewing to activity when gaze is cued to the eye-region of facial stimuli. For example, Hadjikhani et al. (2017a, 2017b) found that participants on the autism spectrum had greater activation of the amygdala and superior colliculus than neurotypical participants when gaze was cued to the eye region of facial stimuli, but equivalent activation for free viewing—suggesting eye gaze plays a causal role in subcortical hyperactivation in ASD. In contrast to Lassalle et al. (2017), participants on the autism spectrum showed greater amygdala activation than neurotypical participants in response to all facial expressions when gaze was cued to the eyes, including happy expressions, although the effect was greatest for fear. These mixed findings may result from use of different stimuli or analytic techniques. Where Lassalle et al. used static faces, Hadjikhani et al. presented short movies of faces morphing from a neutral to emotional expression. Evidence suggests that neural responses differ for dynamic and static facial stimuli (Kilts et al., 2003; Pitcher & Ungerleider, 2021; Trautmann et al., 2009). Furthermore, Hadjikhani et al. used a fixation baseline for determining neural activity, whereas Lassalle used neutral faces. Given evidence that neutral faces may generate excessive amygdala activity in people on the autism spectrum (Hadjikhani et al., 2017a, 2017b; Tottenham et al., 2014), using a neutral baseline may underestimate their amygdala response to emotional faces. Hadjikhani et al. concluded that people on the autism spectrum show an over-reactive subcortical response not only to threat-related, but also socially motivating stimuli. They speculated that an overreactive amygdala and consequent over-sensitivity to emotions may be the cause of eye avoidance in ASD.

Tottenham et al. (2014) used a combination of eye tracking and fMRI to measure visual fixation and amygdala responsiveness to angry and neutral facial stimuli during free viewing and when gaze was cued to the eye region. During free viewing, participants on the autism spectrum were less likely to direct gaze toward the eye region of neutral, but not angry faces. While Tottenham et al. found amygdala activity was greater in participants on the autism spectrum than neurotypical controls for both the free viewing and experimental conditions, gaze manipulation magnified this difference in the case of neutral, but not angry faces. Importantly, increases in amygdala activity during gaze manipulation were greatest for participants who directed their gaze least to the eyes of faces during free viewing. The authors concluded that eye contact causes a heightened emotional response for people on the autism spectrum and that eye avoidance is a strategy used to reduce amygdala-related arousal.

Interestingly, Tottenham et al. (2014) found that participants on the autism spectrum who made more eye movements toward the eye region of facial stimuli during free viewing rated faces as less threatening, and this effect was largest for neutral faces. Participants on the autism spectrum also made significantly more errors in identifying the emotions of neutral faces, with a tendency to misinterpret them as showing negative emotions, such as fear or anger. The authors interpreted these findings as representing a negativity bias for ambiguous stimuli, in line with previous findings in ASD (Kuusikko et al., 2009). Tottenham et al.’s findings suggest gaze avoidance may be related to misapprehension of threat in people on the autism spectrum. Importantly, amygdala activity during gaze manipulation was positively correlated with threat ratings for neutral faces. This effect fully mediated group differences in emotion recognition, suggesting amygdala hyperactivity interferes with face processing and directly contributes to a negativity bias in people on the autism spectrum.

Zürcher et al. (2013a, 2013b) investigated neural activation when people on the autism spectrum attempted to identify Thatcherised faces. Thatcherised faces have inverted eyes and/or mouths, which appear unremarkable to most observers when presented inverted, but grotesque when presented upright (Thompson, 1980). Zürcher, Donnelly, et al. presented pairs of inverted or upright faces to participants, who were asked to identify which face was Thatcherised. In participants on the autism spectrum, cuing gaze to the eyes, as opposed to the mouth, improved the ability to discriminate Thatcherised from typical faces. Furthermore, participants on the autism spectrum, but not neurotypical participants, showed activity in the subcortical pathway, including the amygdala, pulvinar, and superior colliculus, when gaze was cued to the eye region of upright faces. Like Tottenham et al. (2014), Zürcher, Donnelly et al. speculated that amygdala hyperactivity may be related to misinterpretation of threat during perception of ‘grotesque’ stimuli and that threat bias may underlie eye avoidance in ASD.

While most studies have used an experimental manipulation in which gaze was cued to the eye region for the duration of the stimuli presentation, Kliemann et al. (2012) used a novel paradigm. Specifically, Kliemann et al. manipulated participants’ starting fixation on facial stimuli (eye or mouth region) and measured subsequent eye movements and amygdala activity using a combination of eye tracking and fMRI. Participants on the autism spectrum had elevated amygdala responses compared to neurotypical participants when starting on the eye region, while neurotypical participants had elevated amygdala responses compared to participants on the autism spectrum when starting on the mouth region. Furthermore, participants on the autism spectrum made more eye movements away from the eye region and neurotypical participants made more eye movements towards the eye region. Importantly, in participants on the autism spectrum, there was a positive correlation between amygdala activity and frequency of subsequent eye movements away from the eye region—suggesting amygdala activity is involved in eye avoidance, not eye fixation, in this population.

To date, most studies have provided evidence supporting the eye avoidance hypothesis. However, there are three notable exceptions. Perlman et al. (2011) used fMRI to measure amygdala activation while participants viewed faces freely or according to three different scan paths in which the duration of focus on the eyes was low (32%), medium (48%) or high (56%). Participants followed the scan paths by tracking a red crosshair as it jumped to new locations on the facial stimuli every 500 ms. Participants on the autism spectrum showed significantly less amygdala activity than neurotypical participants during free-viewing. However, Perlman et al. found no increase in amygdala activity during the experimental manipulations in participants on the autism spectrum.

Perlman et al.’s (2011) contradictory results may be a product of methodological differences. Where Perlman et al. manipulated the duration of time participants spent looking at the eye region according to different scan paths, other studies constrained gaze entirely to the eye region (Hadjikhani et al., 2017a, 2017b; Lassalle et al., 2017; Tottenham et al., 2014). Notably, amygdala activity in the control group declined during the gaze manipulations, such that there was no difference between participants on the autism spectrum and neurotypical controls. Given the amygdala’s role in directing attention to salient stimuli (Kliemann et al., 2012), Perlman et al. speculated that the decline in activation among control participants occurred because there was no need for the amygdala to direct attention during the gaze manipulation. Other studies, however, have found that amygdala activity in neurotypical participants does not change when gaze is cued to the eyes using a fixation cross (Hadjikhani et al., 2017a, 2017b). Given that attention can modulate amygdala activity (Klumpp et al., 2012), it possible the instruction to follow a crosshair reduced activation during the gaze manipulations because participant attention was focussed on the crosshair, rather than the facial stimuli.

Other studies providing evidence against the eye avoidance hypothesis have used fixation crosses to direct gaze to the eye region. For example, Dapretto et al. (2006) measured neural responses in neurotypical controls and individuals on the autism spectrum while they imitated or observed emotional faces. Like Perlman et al. (2011), Dapretto et al. found that people on the autism spectrum had equivalent amygdala activity to neurotypical participants. Meanwhile, Davies et al. (2011) presented negatively valanced facial stimuli with averted or direct gaze to children on the autism spectrum and neurotypical controls. A fixation cross presented prior to the facial stimuli was used to cue gaze to the eye region. Davies et al. found that neurotypical participants had increased activation of the amygdala and associated subcortical regions when viewing negatively valanced facial stimuli with direct gaze. However, participants on the autism spectrum did not show significantly increased activity in these subcortical regions. Davies et al. concluded that social-emotional processing difficulties in ASD are related to reduced salience of social stimuli, rather than eye avoidance. Importantly, however, between subject analyses showed no difference in subcortical activity between participants on the autism spectrum and neurotypical controls. As with Dapretto et al. and Perlman et al., Davies et al.’s results suggest that participants on the autism spectrum and neurotypical controls experience equivalent amygdala activity when gaze is cued to the eyes of facial stimuli. Thus, support for the amygdala theory of autism is questionable, based on these results.

Given amygdala response may vary by emotional expression, studies that test neural responses to combinations of emotions (e.g., Dapretto et al., 2006; Davies et al., 2011) may not provide a sufficiently granular approach to understanding the amygdala response to eye gaze—potentially, explaining some of the mixed findings in this review. While there is some evidence that emotional expression does not influence the magnitude of amygdala response to eye gaze in participants on the autism spectrum (i.e., Dalton et al., 2005; Kliemann et al., 2012), other studies have found that amygdala response varies by emotion. For example, Hadjikhani et al. (2017a, 2017b) found that amygdala response to eye gaze was greatest for fearful faces in participants on the autism spectrum. Meanwhile, Tottenham et al. (2014) found a substantial increase in amygdala response to neutral faces among participants on the autism spectrum during gaze manipulation, but no increase in response to angry faces. Finally, Lasalle et al. (2017) found participants on the autism spectrum had increased amygdala response to low intensity fearful faces, but decreased response to happy faces compared to neurotypical participants. The gaze direction of facial stimuli also appears to modulate amygdala response to faces in ASD. For example, participants on the autism spectrum tend to exhibit overactivity in the amygdala and/or associated subcortical regions in response to fearful faces with direct gaze (Hadjikhani et al., 2017; Hadjikhani et al., 2017a; Lassalle et al., 2017; Zürcher et al., 2013a, 2013b). However, the subcortical response to fearful faces with averted gaze is attenuated, even when participant gaze is directed to the eye region (Zürcher et al., 2013a, 2013b). Based on this evidence, gaze direction and type of emotional expression appear to be important when considering the role of eye gaze in ASD.

In summary, this review provides evidence against the amygdala theory of autism. Specifically, none of the reviewed studies observed reduced amygdala activity in participants on the autism spectrum compared to neurotypical controls when gaze was cued to the eyes. A recent meta-analysis of face processing studies (based predominantly on free-viewing paradigms) has reported that the primary difference between participants on the autism spectrum and neurotypical controls is amygdala hypoactivation in the participants diagnosed with ASD. The findings of the current review suggest these results may be confounded by gaze preferences among participants on the autism spectrum—highlighting the importance of controlling for gaze in future face processing studies.

Meanwhile, eight of eleven studies reviewed support the eye avoidance hypothesis. Two studies have shown that amygdala activity is positively associated with visual attention to the eye region in participants on the autism spectrum, but not neurotypical controls (Dalton et al., 2005). Three studies found that amygdala activity and/or activity in the subcortical threat detection system was increased in participants on the autism spectrum compared to control participants when gaze was directed to the eye region of fearful or ‘grotesque’ faces (Lassalle et al., 2017; Zürcher et al., 2013a, 2013b). Meanwhile, studies that manipulated gaze and compared neural responses to free-viewing suggest eye gaze may be a direct cause of amygdala hyperactivity in ASD (Hadjikhani et al., 2017a, 2017b; Tottenham et al., 2014). Finally, amygdala activity precedes subsequent gaze away from the eye region of facial stimuli in people on the autism spectrum—indicating eye avoidance is a strategy used to manage amygdala-mediated over-arousal (Kliemann et al., 2012). Interestingly, effects were most consistent for neutral and fearful faces, including faces expressing low-intensity fear (Hadjikhani et al., 2017a, 2017b; Lassalle et al., 2017; Tottenham et al., 2014), suggesting threat sensitivity or negativity bias may be implicated in eye avoidance in ASD. Importantly, eye avoidance is likely to reduce opportunities for social learning among children on the autism spectrum, with cascading implications for development of the social brain (Klin et al., 2020; Shultz et al., 2018).

## Eye Gaze and Activity in the ‘Social Brain’

It has been proposed that amygdala activation in response to eye contact influences and is influenced by the ‘social brain’, including cortical regions such as the fusiform gyrus, superior temporal sulcus, temporoparietal junction, medial prefrontal cortex and inferior frontal gyrus (Blakemore, 2008; Senju & Johnson, 2009). More specifically, eye contact elicits activity in subcortical regions, including the amygdala, which activates wide ranging cortical areas. In turn, cortical inhibitory systems play an important role in modulating amygdala responses to eye contact (Senju & Johnson, 2009; Skuse, 2003). Indeed, a recent study with neurotypical participants has shown that cueing gaze to the eye region of facial stimuli increases amygdala connectivity with the ‘social brain’ and significantly increases activity in these regions (Hadjikhani, Åsberg Johnels, et al., 2017; Hadjikhani, Zurcher, et al., 2017). This section reviews studies that measured the relationship between eye gaze and neural responses in the social brain among participants on the autism spectrum—identifying areas of the social brain that are ‘normalised’ by eye gaze and areas that continue to exhibit atypical activity.

### The Fusiform Face Area

The fusiform face area (FFA), in the lateral part of the fusiform gyrus, is a key region of the social brain involved in visual perception of faces. The FFA selectively responds to faces over other types of objects (Kanwisher et al., 1997; Puce et al., 1995) and its response is increased by looking at eyes (Morris et al., 2007). There is some evidence that people on the autism spectrum demonstrate FFA hypoactivity when viewing images of faces (Perlman et al., 2011). However, two quantitative meta-analyses found that FFA activity does not differ between participants on the autism spectrum and neurotypical participants (Costa et al., 2021; Samson et al., 2012), with Samson et al. citing mixed findings and divergent methodologies. One methodological difference that may account for contradictory findings in previous studies is the duration of time that participants spend looking at the eyes of facial stimuli.

Indeed, evidence suggests there is a positive association between eye gaze and FFA activity in participants on the autism spectrum (Dalton et al., 2005). Furthermore, studies have found FFA activity in participants on the autism spectrum does not differ from neurotypical participants when cued to the eye region of facial stimuli (Dapretto et al., 2006; Davies et al., 2011; Zürcher et al., 2013a, 2013b). Finally, Perlman et al. (2011) found that FFA activity was significantly reduced in participants on the autism spectrum during free viewing, but more closely approximated that of controls with increasing duration of eye gaze. These results suggest that eye avoidance plays a causal role in FFA hypoactivation associated with ASD. Importantly, however, these findings may not extend to faces with averted gaze, with evidence suggesting participants on the autism spectrum show less FFA activity than control participants for fearful faces with averted, as opposed to direct gaze (Zürcher et al., 2013a, 2013b).

### The Superior Temporal Sulcus and Temporoparietal Junction

Other key social brain regions are the superior temporal sulcus (STS) and temporoparietal junction (TPJ) in the temporoparietal cortex, which are involved in processing of gaze and face movement (Haxby & Hoffman, 2000; Pitcher & Ungerleider, 2021; Puce et al., 1998), and have been linked to perspective-taking (Jackson et al., 2006; Pitcher & Ungerleider, 2021; Ruby & Decety, 2003) and theory of mind (Saxe & Kanwisher, 2003). Studies have found evidence of STS/TPJ hypoactivity in participants on the autism spectrum when viewing fearful faces (Kim et al., 2015) and for facial stimuli with direct, but not averted, gaze (Pitskel et al., 2011; von dem Hagen et al., 2014). Interestingly, however, Lassalle et al. (2017) found no differences in STS/TPJ activity between participants on the autism spectrum and neurotypical participants when gaze was cued to the eye region of facial stimuli—suggesting that eye avoidance may also be implicated in previous findings of STS/TPJ hypoactivity. Lassalle et al. concluded that looking at the eye region modulates STS/TPJ activity in people on the autism spectrum such that it is resembles that of controls. They hypothesised that improving eye gaze might assist with associated social cognitive functions, such as perspective-taking. To date, however, Lasalle et al.’s is the only study to specifically investigate STS/TPJ activation during a gaze cuing paradigm. Furthermore, their study did not compare experimentally manipulated gaze to a free-viewing condition. Replications using an experimental paradigm with a free-viewing control are necessary to confirm Lasalle et al.’s findings and establish causality.

### The Medial Prefrontal Cortex

The medial prefrontal cortex (mPFC) is a key structure in the social brain implicated in regulation of emotion (Hänsel & von Känel, 2008) and theory of mind (Hartwright et al., 2014). Consistent evidence from free viewing paradigms suggests participants on the autism spectrum experience mPFC hypoactivity during emotion processing and mentalising tasks (Castelli et al., 2002; Kana et al., 2016; Wang et al., 2007). However, Zürcher et al.’s (2013a, 2013b) gaze cuing study found that directing attention to the eye region of upright Thatcherised stimuli led to equivalent activation of the mPFC among participants on the autism spectrum and controls. Further research comparing gaze cuing to a free-viewing control condition are necessary to confirm these findings and establish causality. Importantly, the mPFC may be under-connected to the amygdala for certain facial stimuli, even when gaze is directed to the eye region. Specifically, Lasalle et al. (2017) found a strong positive correlation between activity in the ventromedial PFC (vmPFC) and the amygdala in neurotypical participants when viewing fearful faces that was absent in participants on the autism spectrum.

Lasalle et al.’s (2017) findings corroborate other evidence of reduced connectivity between the vmPFC and the amygdala in people on the autism spectrum (Li et al., 2021; Swartz et al., 2013). Amygdala-mediated arousal elicited by eye contact is inhibited by prefrontal feedback in neurotypical people (Skuse, 2003). Strong medial prefrontal-amygdala connectivity, in particular, is necessary for amygdala inhibition (Kim et al., 2011) and habituation (Hare et al., 2008; Swartz et al., 2013). Interestingly, several studies have reported decreased amygdala habituation in response to facial stimuli in ASD (Kleinhans et al., 2009; Lombardo et al., 2009; Swartz et al., 2013), with habituation to neutral faces correlating negatively with social difficulties (Swartz et al., 2013). Lassalle et al.’s findings suggest that ASD may be characterised by over-sensitivity to low-intensity fear, relative to neurotypicals. In the context of reduced prefrontal inhibition, the authors speculate that people on the autism spectrum compensate by using eye avoidance to manage strong emotional responses. Interestingly, substantial evidence suggests that children on the autism spectrum often have difficulty with emotional regulation, which is linked to aggression and temper tantrums, as well as anxiety and depression in this population (Mazefsky et al., 2013; Samson et al., 2015). Atypical connectivity between the amygdala and the vmPFC may play a role in emotional regulation difficulties in ASD.

### The Inferior Frontal Gyrus

The inferior frontal gyrus (IFG), is another prefrontal region of the social brain. It is implicated in emotion perception and regulation (Carr et al., 2003; Chick et al., 2020; Wager et al., 2008), as well as the ability to use information from the eyes to perceive mental states (Dal Monte et al., 2014). In a gaze cuing paradigm, Davies et al. (2011) found that participants on the autism spectrum showed less activity than neurotypical controls in ventral areas of the IFG when viewing negatively valanced (angry and fearful) faces.^2^ While neurotypical controls activated these regions of the IFG when viewing negatively valanced faces with direct, but not averted gaze, participants on the autism spectrum did not activate these brain regions in either condition. Given direct gaze conveys social information with immediate relevance to the individual, Davies et al. speculate that hypoactivation of the IFG may represent difficulty comprehending the communicative intent of gaze direction. Unlike Davies et al., Zürcher et al. (2013a, 2013b) observed robust activation of the IFG when participants on the autism spectrum viewed fearful faces with direct compared to averted gaze. Contradictory results in these studies may be the result of testing neural responses to different combinations of facial expressions. Where Davies et al. assessed neural responses to a combination of angry and fearful faces, Zürcher et al. assessed fearful faces individually. Interestingly, Davies et al. found that time fixating the eye region was not associated with IFG activity in participants on the autism spectrum, but increasing proportions of gaze toward the eyes relative to other facial features was associated—leaving open the possibility that eye gaze may be related to IFG activity in ASD. Gaze manipulation studies are necessary to more rigorously examine this possibility.

The dorsal region of the IFG, known as the pars opercularis, is an important node in the brain’s mirror system (Carr et al., 2003). Mirror neurons are active during action observation as well as imitation and are proposed to facilitate interpretations of the intentions of others (Rizzolatti & Fabbri-Destro, 2010). There is some evidence that participants on the autism spectrum show attenuated activity in the dorsal IFG, even when gaze is cued to the eye region of facial stimuli. For example, Dapretto et al. (2006) found that participants on the autism spectrum showed hypoactivation of the pars opercularis when imitating and observing emotional faces. According to mirror system models of empathy, when people view emotional expressions, action representations are relayed from the pars opercularis to the anterior insula, which interfaces with limbic regions to translate these representations into felt experience (Carr et al., 2003; Ferrari et al., 2017; Jabbi & Keysers, 2008). Interestingly, Dapretto et al. observed insula hypoactivity in combination with attenuated activity in the pars opercularis when participants on the autism spectrum imitated and observed emotional faces. Furthermore, activity in both the insula and IFG was positively associated with social functioning, as defined by higher scores on the social subscales of the Autism Diagnostic Observation Schedule—Generic and the Autism Diagnostic Interview—Revised. Dapretto et al. speculated that hypoactivity of these brain regions is associated with empathising difficulties in ASD, in line with the *broken mirror* theory of autism (Ramachandran & Oberman, 2006).

Importantly, however, insula and IFG hypoactivity are not universally observed in participants on the autism spectrum when gaze is cued to the eyes. Instead, activity in these regions appears to be modulated by a number of factors. For example, Zürcher et al. (2013a, 2013b) observed reduced activity in the pars opercularis and anterior insula compared to controls when participants on the autism spectrum discriminated inverted Thatcherised faces, but not upright faces. Lassalle et al. (2017) observed IFG and insula hypoactivity when participants on the autism spectrum viewed faces with happy expressions, but not fearful expressions. Finally, Zürcher et al. (2013a, 2013b) found that participants on the autism spectrum had attenuated activity compared to controls in the pars opercularis and anterior insula when cued to the eye region of fearful faces with averted gaze. When viewing fearful faces with direct gaze, however, participants on the autism spectrum exhibited robust activity in fronto-insular regions. Importantly, a recent free-viewing study noted equivalent activation of areas involved in affective empathy, including the IFG and insula, when participants on the autism spectrum and neurotypical controls viewed videos of people in pain (Hadjikhani et al., 2014). These finding suggest mirror regions are activated in ASD when viewing others in pain, even when gaze is not cued to the eye region. Clearly, further research is necessary to better understand factors that modulate neural activity in these regions in ASD.

### Social Brain Summary and Implications

In summary, the gaze cuing studies reviewed here suggest that directing gaze to the eye region of faces may modulate activity in key areas of the social brain, including the mPFC, STS/TPJ and FFA, such that patterns of activation in participants on the autism spectrum resemble those of controls (Perlman et al., 2011; Zürcher et al., 2013a, 2013b). These results indicate that eye avoidance may play a role in mPFC, STS/TPJ, and FFA hypoactivation in ASD and suggest that interventions to improve eye gaze may have flow-on benefits for social-cognitive functioning in children on the autism spectrum (Lassalle et al., 2017). The results also highlight the importance of controlling for gaze in future research on neural differences in ASD. It should be noted, however, that only one of the studies reviewed in this section compared gaze manipulation to free viewing (Perlman et al., 2011). Future research is therefore necessary to clearly establish that reduced eye gaze plays a causal role in hypoactivation of these brain regions.

Importantly, some brain regions show atypical patterns of activity in ASD, even in gaze cuing paradigms. Specifically, participants on the autism spectrum show reduced connectivity between the amygdala and vmPFC when viewing fearful expressions—suggesting reduced prefrontal regulation of amygdala-mediated arousal responses may be implicated in eye avoidance (Lassalle et al., 2017). Furthermore, the insula and inferior frontal regions exhibit hypoactivity for certain facial stimuli, even in gaze cueing paradigms (Dapretto et al., 2006; Davies et al., 2011; Zürcher et al., 2013a, 2013b). Importantly, however, hypoactivity in these brain regions is not universally observed. Instead, it is evident in response to faces with averted gaze (Zürcher et al., 2013a, 2013b), happy faces (Lassalle et al., 2017) and in studies that tested neural response to combinations of emotional expressions (Dapretto et al., 2006; Davies et al., 2011). Future research is necessary to understand factors that modulate responses to eye gaze in these fronto-insular regions.

## Future Directions

Findings from this review provide evidence in support of the eye avoidance hypothesis. Most importantly, gaze cuing studies have shown that people on the autism spectrum experience amygdala hyperactivity for certain facial stimuli, including faces with direct gaze and fearful, neutral or ambiguous expressions. Furthermore, directing attention to the eye region of faces appears to normalise activity in certain areas of the social brain in ASD, although studies comparing gaze manipulation to free viewing are necessary to confirm that reduced eye gaze plays a causal role in hypoactivation of these regions. As a matter of priority, more gaze cuing research is needed to confirm the findings of this review, given the small sample of studies considered. Moreover, given studies included in this review used samples consisting predominantly of males with higher functioning ASD, future studies should be conducted using more varied samples in order to understand factors that might moderate the relationship between neural activity and eye gaze. Notwithstanding, the findings of this review provide a strong argument that future research should carefully control for participant gaze, as eye avoidance is likely to confound experimental scenarios (Lassalle et al., 2017; Morris et al., 2007).

Some important questions remain under-investigated. Firstly, many authors have speculated that eye avoidance is driven by amygdala-mediated arousal; to date, however, no study has directly tested whether cueing gaze to the eye region increases physiological arousal in people on the autism spectrum, relative to free viewing. Research using direct measures of autonomic arousal, for example, pupillometry or heart rate variability, is necessary to test this hypothesis (Dalton et al., 2005). In addition, gaze manipulation studies with free viewing control conditions have tended to focus on discrete ROIs (Hadjikhani et al., 2017a, 2017b; Kliemann et al., 2012; Tottenham et al., 2014). ROI approaches have been criticised on the basis of increasing false positives and poor replicability (Gentili et al., 2019, 2021). Furthermore, widespread differences in brain activity have been reported when people on the autism spectrum view faces (Lassalle et al., 2017; Zürcher et al., 2013a, 2013b) that cannot be identified using ROI approaches. Future research comparing whole brain responses in gaze cuing and free viewing conditions is necessary to better understand neural responses to eye gaze in the social brain (Gentili et al., 2019, 2021). Importantly, focussing on activity in discrete brain regions neglects the importance of interactions within the neural system (Betzel, 2022). Future studies addressing whole brain connectivity using systems neuroscience methodologies are clearly necessary to better understand mechanisms responsible for eye avoidance in ASD, including the potential role of neural networks and brain hubs for information transfer (Johnson et al., 2021).

Research comparing neural responses to different types of facial stimuli, including dynamic vs static faces, faces with varying gaze direction, and faces with different emotional expressions, is also necessary to better understand the response to eye gaze in ASD. Importantly, participants on the autism spectrum have hypoactivity in a wide variety of brain regions when they view fearful faces with averted compared to direct gaze, even when their own gaze is cued to the eye region of facial stimuli (Zürcher et al., 2013a, 2013b). Given most gaze cuing research to date has used facial stimuli with direct gaze, it will be important for future studies to disentangle how neural responses vary in relation to gaze direction. Use of dynamic stimuli in future research may also be important, given dynamic face processing activates different neural pathways from static tasks (Kilts et al., 2003; Pitcher & Ungerleider, 2021; Trautmann et al., 2009) and people on the autism spectrum show more pronounced reductions in eye fixation and brain activity when viewing dynamic scenes (Sato et al., 2012; Speer et al., 2007). While there is some evidence that people on the autism spectrum experience hyperactivity in key regions of the social brain when viewing fearful faces, but hypoactivity for happy faces (Lassalle et al., 2017), gaze cuing studies have not yet provided consistent findings regarding neural responses to socially rewarding expressions in ASD. Research to clarify activation patterns in response to varying emotional expressions may help to integrate arousal-based and social motivation theories of ASD.

Given evidence that ASD represents the culmination of an atypical developmental pathway, understanding its aetiology is best advanced through prospective longitudinal research initiated in infancy (Constantino et al., 2021; Johnson et al., 2021). However, the vast majority of research, including the papers reviewed here, use between-subjects approaches to identify differences between people with and without diagnoses of ASD. Longitudinal eye-tracking studies commencing prior to ASD diagnosis may help to better understand the relationship between autonomic arousal, eye gaze and the development of social communication difficulties. For example, a recent study showed that infants at risk of ASD have greater pupillary arousal responses to emotional faces, with these responses predicting greater attention to faces at 9 months of age, but reduced attention to faces and lower social functioning at 18 months of age (Wagner et al., 2016). Additional longitudinal research is necessary to determine if hyperarousal in response to faces is an inherited physiological trait with downstream consequences for eye avoidance and social development or, instead, a consequence of ASD itself.

Although preferential gaze to the eye region is highly heritable (Constantino et al., 2017), infants later diagnosed with ASD cannot be reliably differentiated from typically developing infants on the basis of eye gaze until 12 months of age (Rozga et al., 2011; Zwaigenbaum et al., 2005). In fact, at six months of age, infants later diagnosed with ASD may show greater preference for looking at faces, and the eyes within faces, than their typically developing peers (Elsabbagh et al., 2013; Ozonoff et al., 2010). However, eye contact in these infants declines significantly between 2 and 24 months of age (Jones & Klin, 2013; Ozonoff et al., 2010), with steeper declines associated with more severe social difficulties (Jones & Klin, 2013). A declining trajectory of eye contact during the first two years of life suggests that genetic liabilities are not sufficient to cause reductions in eye gaze, but instead interact with environmental factors in an iterative fashion (Constantino et al., 2017). Furthermore, morphological studies indicate that amygdala volume also changes over time in people on the autism spectrum—with increased volumes in toddlers and children and decreased volumes in adolescents and adults (Bellani et al., 2013). It is currently unknown when in development amygdala overgrowth and atrophy occurs. However, both volume increases in children and reductions in adults have been associated with social difficulties (Bellani et al., 2013). In order to understand ASD’s aetiology, longitudinal studies are necessary to track how environmental and neurobiological factors interact to reduce or maintain eye gaze (Constantino et al., 2017; Jones & Klin, 2013).

Importantly, this future research may help to uncover protective factors that could be augmented through early intervention (Constantino et al., 2021; Klin et al., 2020). There is emerging evidence that interventions commencing during the first 2 years of life, when the first signs of atypical development are observed and the brain is rapidly developing, may lead to a beneficial impact on developmental outcomes in later childhood (Carter et al., 2011; Green, 2020; Kasari et al., 2014; Rogers et al., 2012; Whitehouse, 2017). For example, a recent randomised controlled trial found that the delivery of a social communication intervention to infants showing early signs of ASD significantly reduced the likelihood of them receiving a diagnosis of ASD at age three years (Whitehouse et al., 2019, 2021). Given gaze cuing normalises activity in some areas of the social brain, it is plausible that social attention therapies may also accentuate activity in these areas, with potential benefits for social communication in children on the autism spectrum. Indeed, a recent study has shown that social cognition training increases activity in key regions of the social brain (amygdala, mPFC, and insula) in children on the autism spectrum during social perception tasks (Ibrahim et al., 2021). Importantly, increases in mPFC activity were associated with increases in social functioning, as measured by the Social Responsiveness Scale (Ibrahim et al., 2021). Greater understanding of the cognitive and biological underpinnings of differences in eye contact will provide insight into the developmental pathways contributing to ASD, and how tailored interventions can be provided to infants and children to support their long-term development.

Finally, ASD is a highly heterogeneous diagnostic category (Waterhouse et al., 2016), in which diverse neurobiological factors may result in common endophenotypes (Constantino et al., 2021; Jones & Klin, 2013). In fact, Joseph et al. (2008) reported substantial differences in arousal responses to facial stimuli in participants on the autism spectrum. It is possible that reduced eye gaze has different aetiologies, with excessive arousal the defining feature of one particular pathway (Joseph et al., 2008). Moreover, as several authors note, reduced reward and/or increased arousal in response to eye contact are not mutually exclusive and may both influence the expression of ASD (Cuve et al., 2018; Kaartinen et al., 2012; Kliemann et al., 2012). Specifically, Kliemann et al. (2012) and Cuve et al. (2018) have proposed that ASD should be dimensionally conceptualised, with different contributions of reduced orientation or increased avoidance contributing to its manifestation. Longitudinal studies specifically measuring these indices may provide clarity on this matter.

In addition to the possibility that the aetiology of reduced eye gaze in people on the autism spectrum is heterogeneous, it is also possible that eye gaze itself differs between individuals, as has been reported for other social differences, such as difficulty with expression recognition (Loth et al., 2018). In support of this possibility, findings from eye tracking studies measuring gaze in people on the autism spectrum have been mixed. A recent review by Cuve et al. (2018) reported that, of sixteen eye tracking studies, nine reported reduced eye fixation in participants on the autism spectrum compared with controls; however, five reported no difference in eye fixation. It is possible that individual differences across different experimental samples contributed to differences in study findings. One source of individual differences may be varying levels of alexithymia between individuals on the autism spectrum (Bird et al., 2011). Alexithymia is a personality trait which involves difficulties in identifying and describing one’s own emotions, in addition to externally oriented thinking (Taylor & Bagby, 2000). Alexithymia is also associated with difficulties identifying the emotions of others (Cook et al., 2013). The ‘alexithymia hypothesis’ suggests that emotional difficulties that can be experienced by people on the autism spectrum might in fact reflect co-occurring alexithymia, rather than ASD per se (Bird & Cook, 2013).

As with ASD, higher levels of alexithymia have been linked to reduced eye gaze (Bird et al., 2011; Cuve et al., 2021). In fact, research with participants on the autism spectrum suggests alexithymia may be a stronger predictor of attenuated eye gaze than ASD itself (Bird et al., 2011; Cuve et al., 2021). Interestingly, the response to eye gaze may vary among people on the autism spectrum, based on their level of alexithymia. In participants on the autism spectrum, for example, gaze away from the eye region of facial stimuli has been associated with less accurate emotional recognition (Kliemann et al., 2012). However, in people with high levels of alexithymia, increased eye fixation has been associated with less accurate emotional recognition (Fujiwara, 2018). While eye gaze appears to normalise activity in many regions of the social brain for people on the autism spectrum, eye fixation is associated with decreases in social brain activity among people with high levels of alexithymia (Zimmermann et al., 2021). Future research should attempt to disentangle the interplay between alexithymia, neural response to eye gaze, and features of ASD.

## Conclusion

In conclusion, reduced eye contact is an important endophenotype that may play a role in the development of ASD. To explain the mechanisms underpinning eye contact differences, it is often argued that faces and eyes lack salience for people on the autism spectrum. In fact, since autism was first described by Kanner (1968), there has been an assumption that lack of eye contact in children on the autism spectrum signals social indifference. However, recent research suggests this assumption may be far from the truth for at least some people on the autism spectrum. Studies that have directly measured the relationship between neural activity and eye gaze indicate that eye avoidance is a strategy used to reduce amygdala-related hyperarousal for people on the autism spectrum. These studies suggest that previous findings of hypoactivity in many regions of the social brain in ASD may be the result of eye avoidance—highlighting the importance of controlling for gaze in future studies.

Rather than being apathetic, people on the autism spectrum may instead be hypersensitive to certain social stimuli and what appears to be indifference, may in fact, reflect avoidant behaviour (Lassalle et al., 2017). Importantly, the eye avoidance hypothesis appears more consistent with the lived reality of many people on the autism spectrum, who often report that eye contact is a source of stress and anxiety (Trevisan et al., 2017). As one respondent to an online survey stated, “… eye contact triggers a fight or flight response so strong that it overrides everything else …” (Trevisan et al., 2017, p. 8). Longitudinal studies should be prioritised to determine if this hyperarousal is an inherited physiological trait or a consequence of ASD itself. Arousal responses may prove an important thread to follow in the effort to understand how eye avoidance develops in ASD and how its developmental consequences might be mitigated.

## Footnotes

1 Note that the terms used in the literature sometimes confound constructs. Specifically, visual attention is not the same as visual fixation because it is possible to covertly attend to someone’s eyes without looking at the eye region and conversely covertly attend elsewhere while looking at someone’s eyes (Kulke et al., 2021, 2016). This is an important distinction to draw in the context of empirical studies that attempt to manipulate visual attention by controlling gaze direction.

2 Please note that Davies et al. (2011) refer to the ventrolateral prefrontal cortex (vlPFC), where other studies use the term IFG. Both areas refer to the same Brodmann areas (BA44, 45 and 47). In the current paper we use the term IFG.
